# Editorial: Systemic RNA Signalling in Plants

**DOI:** 10.3389/fpls.2022.878728

**Published:** 2022-03-21

**Authors:** Michitaka Notaguchi, Vicente Pallas, Jie Qiu, Xutong Wang

**Affiliations:** ^1^Bioscience and Biotechnology Center, Nagoya University, Nagoya, Japan; ^2^Instituto de Biología Molecular y Celular de Plantas (IBMCP), Consejo Superior de Investigaciones Científicas-Universitat Politécnica de Valencia, Valencia, Spain; ^3^Shanghai Key Laboratory of Plant Molecular Sciences, College of Life Sciences, Shanghai Normal University, Shanghai, China; ^4^Department of Agronomy, Purdue University, West Lafayette, IN, United States

**Keywords:** systemic signaling, small RNA, mRNA, mobile RNA, long-distance communications, biotechnology

Systemic signaling is a fundamental mechanism to orchestrate organ/tissue/cellular behavior in multicellular organisms. In plants, long-distance signaling between distant organs is achieved, in part, through the transport of signaling molecules including RNA species such as miRNA, siRNA, and mRNA, *via* phloem sieve elements, reviewed in Lucas et al. (2001); Kehr and Buhtz (2007); Pallas and Gómez (2013); Notaguchi and Okamoto (2015). This Research Topic “Systemic RNA Signalling in Plants” focuses on the study of the latest bioinformatic and molecular methods applied to identify mobile coding and non-coding RNA loci responsible for systemic regulation of plant development or interactions with and responses to pathogens and environmental stresses. Since many biological questions and experimental challenges exist in this field, gaining knowledge of systemic RNAs will advance our understanding of how plants coordinate heterogenous internal cellular status and external environmental conditions at the whole plant level to increase the fitness of their growth and development. This Research Topic contains two reviews, two original research articles, and one opinion article as follows.

Tang et al. provides a comprehensive review on the current understanding of small RNAs that move systemically. Biogenesis of miRNA, siRNA, and other small RNAs as well as trafficking pathways for each RNA species are classified and summarized. Moreover, the recent discovery of the small RNA delivery pathway *via* extracellular vesicles (EV) from plant to pathogen is presented referring to key original research articles. As one remarkable finding, the potential cross-kingdom transfer of small RNA to animals *via* food is introduced, although this Research Topic remains controversial and inconclusive. The function of these RNA species is described as epigenetic inheritance, cleavage of target mRNA–RNA interference, and translational repression. Among them, it is worthwhile to emphasize the use of small RNA-delivered *via* EV for the control of pathogen infection. Yu et al. addresses another historically controversial Research Topic such as the systemic signaling that controls flowering. Different studies have revealed that the Flowering locus T (FT) protein is a major component of *FT* flowering promotion. However, several groups including the authors' group have observed mobility of *FT* mRNA or *FT* mRNA localization near the plasmodesmata in expressed cells as a related phenomenon of symplasmic transport. This mini review summarizes a series of experiments, such as virus-based system, grafting experiments, and intracellular RNA imaging, and discusses potential non-cell autonomous behavior of *FT* mRNA. In addition, as a potential biotechnological tool, the addition of *FT* mRNA sequence to guide RNA is proposed to overcome virus exclusion from the shoot apical meristem to generate heritable virus-induced gene/genome editing. Since gene editing will be a key technology to improve and accelerate agricultural breeding, such an RNA delivery technique based on an evolutionarily conserved RNA transport system is an attractive catalyst for new opportunities to face the challenges posed by global climate change to sustainable and robust agriculture. Lezzhov et al. contributed an opinion article about phloem exit as a possible control point in selective systemic transport of RNA. According to these authors, this selective transport would rely on the presence of “phloem transport signals” (PTS) that would be bound by PTS-binding proteins involved in selection of RNA molecules for exit to surrounding tissues. Conceivably, the existence of non-identical PTS-binding proteins in different plant organs would explain the fact that distinct subsets of mobile mRNAs are delivered to different target organs. The authors also discuss potential involvement of the endoplasmic reticulum and actin network in RNA transport in sink-to-source manner, opposite the direction of phloem flow. Vargas-Hernández et al. and Calderón-Pérez et al. provide original studies related to this field. Vargas-Hernández et al. provide a study on *ANAC087*, a homolog of *CmNACP1*, the mRNA of which has been shown to move long-distance through the phloem in *Cucurbita maxima* (pumpkin) and through a graft junction. By taking advantage of the model plant system, Arabidopsis, the authors address the function of *CmNACP1* homologs. Out of six Arabidopsis homologs, the authors focused on *ANAC087* because it possesses the highest sequence similarity. The promoter GUS analysis of *ANAC087* showed that strong GUS expression was found in leaf vascular tissue and stem as well as the shoot apex, roots and the base of the trichomes. Although the mobility of *ANAC087* mRNA has not been examined by the authors, the biological relevance to leaf senescence and aerial rosette formation was demonstrated by overexpression of *ANAC087*. Calderón-Pérez et al. demonstrated the potential use of the plant macromolecular trafficking system to mobilize antimicrobial peptides (AMPs). The authors utilized the knowledge that a pumpkin phloem mobile protein, CmPP16, facilitates non-specific RNA transport. It has been thought that CmPP16 regulates plasmodesmata permeability and facilitates the systemic movement of other molecules. The authors identified the citrus homolog, named CsPP16, and engineered transgenic citrus trees harboring CsPP16-fused AMPs. Translocation of the CsPP16-fused AMPs into sieve elements was confirmed in these transgenic citrus plants. Finally, the authors demonstrated the positive effect of the engineering on reduction of live bacterial infection for a period of 1 year. Moreover, further significant reduction was observed when AMPs were combined. This study demonstrated the possible combinational usage of antimicrobials and macromolecular trafficking system to control bacterial infection.

The research articles featured in this Research Topic cover a range of small RNA, mRNA, viral RNA, and components of RNA transport. This Research Topic further advances our understanding of RNA systemic transport and its potential use of bioengineering. Emerging findings and hypothesis need further experimental proofs in future studies. Further studies of RNA trafficking mechanism, such as the molecular forms of trafficking *via* phloem, e.g., RNA-protein complex, the manner of selective transport at the loading and unloading sites, and the cellular translocation pathways between different tissue types, will provide knowledge on their roles in plant biology and also enhance bioengineering techniques for crop improvement.

## Author Contributions

MN drafted the manuscript. VP, JQ, and XW edited and revised the manuscript. All authors contributed to the article and approved the submitted version.

## Conflict of Interest

The authors declare that the research was conducted in the absence of any commercial or financial relationships that could be construed as a potential conflict of interest.

## Publisher's Note

All claims expressed in this article are solely those of the authors and do not necessarily represent those of their affiliated organizations, or those of the publisher, the editors and the reviewers. Any product that may be evaluated in this article, or claim that may be made by its manufacturer, is not guaranteed or endorsed by the publisher.
